# Exploring the Impact of *PARK2* Mutations on the Total and Mitochondrial Proteome of Human Skin Fibroblasts

**DOI:** 10.3389/fcell.2020.00423

**Published:** 2020-06-11

**Authors:** Mara Zilocchi, Ilaria Colugnat, Marta Lualdi, Monica Meduri, Federica Marini, Victor Corasolla Carregari, Mohamed Taha Moutaoufik, Sadhna Phanse, Luisa Pieroni, Mohan Babu, Barbara Garavaglia, Mauro Fasano, Tiziana Alberio

**Affiliations:** ^1^Department of Biochemistry, Research and Innovation Centre, University of Regina, Regina, SK, Canada; ^2^Department of Science and High Technology, Center of Neuroscience, University of Insubria, Busto Arsizio, Italy; ^3^Università Cattolica del Sacro Cuore, Fondazione Policlinico Universitario Agostino Gemelli-IRCCS, Rome, Italy; ^4^Santa Lucia IRCCS Foundation, Rome, Italy; ^5^Unità di Genetica Medica e Neurogenetica, Fondazione IRRCS Istituto Neurologico Carlo Besta, Milan, Italy

**Keywords:** proteomic alterations, interactome, mitophagy, mitochondria, Parkinson’s Disease, Parkin (*PARK2*)

## Abstract

Mutations in *PARK2* gene are the most frequent cause of familial forms of Parkinson’s disease (PD). This gene encodes Parkin, an E3 ubiquitin ligase involved in several cellular mechanisms, including mitophagy. Parkin loss-of-function is responsible for the cellular accumulation of damaged mitochondria, which in turn determines an increment of reactive oxygen species (ROS) levels, lower ATP production, and apoptosis activation. Given the importance of mitochondrial dysfunction and mitophagy impairment in PD pathogenesis, the aim of the present study was to investigate both total and mitochondrial proteome alterations in human skin fibroblasts of *PARK2*-mutated patients. To this end, both total and mitochondria-enriched protein fractions from fibroblasts of five *PARK2*-mutated patients and five control subjects were analyzed by quantitative shotgun proteomics to identify proteins specifically altered by Parkin mutations (mass spectrometry proteomics data have been submitted to ProteomeXchange with the identifier PXD015880). Both the network-based and gene set enrichment analyses pointed out pathways in which Rab GTPase proteins are involved. To have a more comprehensive view of the mitochondrial alterations due to *PARK2* mutations, we investigated the impact of Parkin loss on mitochondrial function and network morphology. We unveiled that the mitochondrial membrane potential was reduced in *PARK2*-mutated patients, without inducing PINK1 accumulation, even when triggered with the ionophore carbonyl cyanide m-chlorophenylhydrazone (CCCP). Lastly, the analysis of the mitochondrial network morphology did not reveal any significant alterations in *PARK2*-mutated patients compared to control subjects. Thus, our results suggested that the network morphology was not influenced by the mitochondrial depolarization and by the lack of Parkin, revealing a possible impairment of fission and, more in general, of mitochondrial dynamics. In conclusion, the present work highlighted new molecular factors and pathways altered by *PARK2* mutations, which will unravel possible biochemical pathways altered in the sporadic form of PD.

## Introduction

Parkinson’s disease (PD) is the second most frequent neurodegenerative disorder after Alzheimer’s disease and is characterized by the loss of dopaminergic neurons in the substantia nigra pars compacta (SNpc). PD is a multifactorial disorder in which both genetic and environmental factors contribute to the onset and progression. As for pathogenetic mechanisms, impairment of the ubiquitin-proteasome system (UPS), increased oxidative stress, dysregulation of protein trafficking, and mitochondrial damage are hallmarks of PD (Schapira and Jenner, 2011; Shi et al., 2017). The co-occurrence of these alterations ultimately leads to the disruption of fundamental cellular processes, thus affecting the maintenance of a correct cellular homeostasis. Only about 5–10% of PD patients suffer from familial forms of the disease, which are characterized by a clear genetic etiology (Shulman et al., 2011).

Over the last 15 years the list of PD-associated genes (*PARK* loci) has grown rapidly (Hernandez et al., 2016). Several mutations have been described to affect the function of these genes, causing both autosomal dominant (e.g., *PARK1*, *PARK8*) and autosomal recessive (e.g., *PARK2*, *PARK7*, *PARK6*) forms of familial PD (Lunati et al., 2018). Of note, most of these genes code for protein products involved in or related to mitochondrial homeostasis, thus highlighting the crucial role of mitochondrial dysfunction in the degeneration of nigral dopaminergic neurons in PD. The *PARK2* gene encodes Parkin, an E3 ubiquitin ligase. Mutations in this gene have been linked to autosomal recessive juvenile PD. This PD form is characterized by an age-of-onset between childhood and 45 years of age (West and Maidment, 2004).

Disease-causing mutations include single base-pair substitutions, small and big (hundreds of thousands of nucleotides) deletions, and splice site mutations. In all cases, mutations lead to a loss of Parkin function, albeit through different mechanisms. This obviously occurs when deletions span several exons. Nonsense-mediated decay would destabilize any truncated transcripts, thus leading to the absence of protein expression. Indeed, there is little evidence that truncated Parkin proteins are expressed in patients with exon deletions. On the other hand, missense mutations appear to cause a loss of Parkin function through decreased catalytic activity and/or aberrant ubiquitination. Point mutations might also cause the destabilization of Parkin, leading to insolubility or rapid proteasomal degradation of the mutant protein (Dawson and Dawson, 2010).

Parkin was described as a molecular factor that plays a fundamental role in mitochondrial dynamics, which is regulated by the interaction between Parkin and PINK1, a serine/threonine kinase, whose mutations are also involved in the development of PD (Geisler et al., 2010). However, the PINK1/Parkin pathway is mostly known for its important role in mitophagy, a quality control process that allows for the degradation of damaged mitochondria (Youle and Narendra, 2011). Under basal conditions, when mitochondrial membrane is properly polarized, PINK1 is imported into the mitochondria, cleaved by several mitochondrial proteases, and rapidly removed through the proteasome. Upon mitochondrial depolarization, the mitochondrial import of PINK1 is inhibited, resulting in its accumulation into the outer mitochondrial membrane (OMM). This process triggers the recruitment of Parkin onto the mitochondrial surface, which, in turn, promotes the ubiquitination of different OMM proteins, thus initiating mitophagy. The impairment of this pathway leads to the accumulation of dysfunctional mitochondria that can contribute to dopaminergic cells death due to lower ATP production, hyperproduction of reactive oxygen species (ROS), and activation of the apoptotic process (Fernández-Moriano et al., 2015).

Although mitophagy impairment may be a leading event in PD pathogenesis, the molecular mechanisms underlying the improper removal of dysfunctional mitochondria are still poorly understood. To fill this gap, we used *PARK2*-mutated primary skin fibroblasts as cellular models to explore the effects of *PARK2* mutations both on the mitochondrial network morphology and on the total and mitochondrial proteome. Skin fibroblasts are an easily accessible peripheral source of proliferating cells. These cells mirror the polygenic risk factor and reflect the cumulative cell damage that occurs in patients (Auburger et al., 2012). A previous study has already shown that fibroblasts derived from *PARK2*-mutated patients can be used to investigate the mitochondrial impairment that characterizes these subjects (Zanellati et al., 2015). For instance, *PARK2*-mutated fibroblasts are characterized by an increased oxygen consumption rate with a reduction in ATP cellular levels, lower mitochondrial membrane potential, and lower complex I activity.

Here, to elucidate the mitochondrial alterations caused by Parkin loss-of-function, we used *PARK2*-mutated primary skin fibroblasts from patients carrying different genetic backgrounds and performed a quantitative shotgun proteomic analyses at the mitochondrial and whole cell proteome levels. Moreover, we analyzed the resulting molecular outcomes, especially on the mitochondrial membrane potential and morphology of the mitochondrial network. Lastly, we identified molecular pathways specifically altered by *PARK2* mutations by performing a network-based analysis.

## Materials and Methods

### Subjects

Primary skin fibroblast cell lines from five *PARK2*-mutated PD patients (two males, three females; mean age 45 ± 20) and five gender- and age-matched controls (two males, three females; mean age 38 ± 13) were obtained from the “Cell line and DNA Bank of Genetic Movement Disorders and Mitochondrial Diseases” of the Telethon Network of Genetic Biobanks (TNGB) (Table 1; Baldo et al., 2016). Personal and clinical data and genetic characterization were collected from patients after specific informed consent. Control subjects gave their consent for research purposes.

**TABLE 1:** Outline of primary skin fibroblast cells from *PARK2*-mutated Parkinson’s disease patients and control subjects.

Subject^a^	Age at onset	Age at skin biopsy	*PARK2* mutations
P1	60–65	70–75	Del_1/p.R275W
P2	15–20	40–45	Del_3-4-5/p.R33X
P3	10–15	15–20	Dup_2/Del_3-4-5
P4	40–45	50–55	p.Q34Rfs × X5 homo
P5	20–25	30–35	p.Thr240Met/Del3
C1	CTRL	25–30	CTRL
C2	CTRL	25–30	CTRL
C3	CTRL	30–35	CTRL
C4	CTRL	50–55	CTRL
C5	CTRL	50–55	CTRL

*^*a*^P, *PARK2*-mutated Parkinson’s disease patients; C, CTRL subjects.*

### Cell Cultures and Treatments

Primary skin fibroblast cell lines were cultured in high glucose Dulbecco’s modified Eagle’s medium (DMEM) (Euroclone) supplemented with 15% (v/v) fetal bovine serum (FBS) (Euroclone), 100 U/ml penicillin, 100 μg/ml streptomycin (Euroclone), and 2 mM L-glutamine (Euroclone) and maintained at 37°C under humidified conditions and 5% CO2. Cells were subcultured twice weekly, detached with Accutase (Euroclone), and centrifuged at 500 × *g* for 10 min at 25°C. Cells were used at passage number lower than 13.

Fibroblast cells were seeded at a density of 5 × 10^5^ per 75 cm^2^ flask for 24 h before treatments. Cells were then exposed to carbonyl cyanide m-chlorophenylhydrazone (CCCP) dissolved in dimethyl sulfoxide (DMSO) at a concentration of 60 μM or to an equal volume of DMSO alone, for 24 h.

### Mitochondrial Enrichment

Mitochondria were isolated from 1.5 × 10^7^ fibroblast cells. After detaching cells with Accutase, mitochondria were isolated using the commercial kit based on surfactants Mitochondrial Isolation Kit MITOISO2 (Sigma-Aldrich), which has been demonstrated to be the best performing method for fibroblasts cells by the mtHPP consortium (Alberio et al., 2017). Briefly, cells were lysed in lysis buffer supplemented with the protease inhibitor cocktail (Sigma-Aldrich) and incubated for 10 min on ice. Two volumes of extraction buffer were added to the lysates before centrifuging at 600 × *g*, for 10 min at 4°C. The supernatant thus obtained was discarded in order to perform a second lysis of the cellular pellet. After a second centrifugation (600 × *g*, 4°C, 10 min), the supernatant was transferred in a fresh tube and centrifuged at 11,000 × *g*, for 10 min at 4°C, to obtain the mitochondrial-enriched fraction. The quality of the mitochondrial enrichment was checked by western blot, by assessing the levels of VDAC1 (a protein of the OMM), CS (a protein of the mitochondrial matrix), and histone H3 (to assess the nuclear contamination) (Supplementary Figure 1).

### Mitochondrial Membrane Potential

Fibroblast cells were seeded in 12-well plates at 8 × 10^3^ cells per well and cultured for 24 h at 37°C in the presence of 60 μM CCCP or an equal volume of DMSO. Culture media were then removed and replaced with fresh DMEM supplemented with 100 nM Mitotracker Red CMXRos (Life Technologies). After 30 min of incubation at 37°C, cells were washed with PBS and fixed with 4% paraformaldehyde for 15 min. Images were acquired through a cooled CCD camera on an Olympus IX81 microscope (40×) and analyzed using the ImageJ software, as previously described (Bondi et al., 2016). Seven fields of view randomly taken from three independent experiments were evaluated for each subject. The image analysis was blinded. Statistical analysis was performed using two-way ANOVA.

### Indirect Immunofluorescence

Fibroblast cells were seeded at a density of 5 × 10^3^ per well onto 18 mm glass coverslips in 12-well plates and cultured for 24 h at 37°C in the presence of 60 μM CCCP or an equal volume of DMSO. Treatments lasted for 24 h. Cells were washed with PBS and fixed with 4% paraformaldehyde for 15 min, permeabilized with Triton X-100 (0.2% Triton X-100 in PBS) for 5 min, and blocked with 5% FBS in PBS for 2 h at RT. Coverslips were incubated overnight at 4°C with the primary antibody against ATP Synthase β (1:400 dilution; A9728, Sigma-Aldrich), RAB7A (HPA006964, 1:200; Sigma-Aldrich), and DRP1 (sc-32898, 1:100; Santa Cruz Biotechnology) in 5% FBS diluted in PBS. Fibroblast cells were then incubated with anti-rabbit Alexa Fluor 488 (1:200 dilution; #AP132JA4; Thermo Fisher Scientific) and anti-mouse Alexa Fluor 647 secondary antibodies (1:200 dilution; A21236, Thermo Fisher Scientific) in 5% FBS in PBS. After mounting coverslips, a laser-scanning confocal microscope (TCS SP5, Leica) with a 63×/1.40 NA oil-immersion objective (HCX PL APO lambda blue) was used. z-stacks with 0.2 μm step size were acquired with sequential excitation at 1024 × 1024 pixels resolution and 1.5× magnification.

### Mitochondrial Network Morphology Analysis

The mitochondrial network morphology analysis was performed on two coverslips from independent biological replicates for each cell line labeled with anti-ATP Synthase β, as described above. Five randomly chosen fields of view were captured and analyzed for each coverslip. The analysis of images was performed using the “Analyze Particles” function of the ImageJ software, as previously described (Bondi et al., 2016). The analysis was blinded. Briefly, the spatial calibration was performed, according to the magnification used during image acquisition. The unsharp mask filter was then applied to all z-stack slices. Then, an automatic threshold (Huang algorithm) was applied to generate a binary image. One-bit images were further processed to remove aberrantly detected objects. The final masks were stored and used to measure several spatial and shape-descriptor parameters among all z-planes using the “Analyze Particles” function, namely Area, Perimeter, Major and Minor axis, Angle, Aspect ratio, Circularity, Roundness, and Solidity.

The distribution density of all parameters was calculated for each field of view. The distance between the empirical distribution density of each sample and the distribution density of all controls/DMSO samples (i.e., particles from five fields of view from each control subject in the DMSO treatment condition) was obtained by the Kolmogorov–Smirnov test. Significant distances were analyzed by two-way ANOVA. All data analysis and statistics procedures were written using the R environment for statistical computing^1^.

The mitochondrial network morphology for the basal, untreated cells was also characterized using the “Analyze Skeleton” function of ImageJ software, as previously described (Valente et al., 2017). First, the images were converted to binary. Binary z-stacks were skeletonized using standard processing operations and the resulting mitochondrial skeleton was analyzed using the “Analyze Skeleton” plug-in, in order to count and measure branches. In detail, the number of branches represents the number of segments that connect endpoints to junctions or junctions to another junction, while the number of junctions is the number of pixels of an object having more than two neighbors. The output tables were compared using the univariate non-parametric Wilcoxon test. The boxplots of control subjects and *PARK2*-mutated patients were generated in R.

### Immunofluorescence Quantification

DRP1 and Rab7A levels were measured in the mitochondrial area defined by the z-projection of ATP Synthase β images in the absence and in the presence of CCCP treatment. The analysis was done using the ImageJ software by setting an automated thresholding process (the IsoData function). Statistical analysis was performed by two-way ANOVA for Rab7A quantification and by two-tailed unpaired *t*-test for DRP1.

### Western Blot Analysis

Cells were lysed in 100 μl RIPA buffer (0.1% SDS, 50 mM Tris–HCl pH 7.6, 150 mM NaCl, 1% sodium deoxycholate, 1% NP-40) supplemented with 1x protease inhibitor cocktail, sheared by ultrasounds, and centrifuged at 15,000 × *g*, for 30 min at 4°C. Proteins were quantified through the BCA assay (Thermo Fisher Scientific). Proteins (20–50 μg) were loaded onto 8% or 16% SDS-PAGE gels and transferred to PVDF membranes at 1.0 mA/cm^2^ for 2 h (TE77pwr; Hoefer). Membranes were blocked with 5% milk powder in tris-buffered saline with 0.05% TWEEN (TBST) for 2 h at RT and then incubated with primary antibodies overnight at 4°C: CS (1:1000 dilution; AMAb91006, Sigma-Aldrich), histone H3 (1:2500 dilution; H0164, Sigma-Aldrich), VDAC1 (1:1000 dilution; ab15895, Abcam), Parkin (1:500 dilution; #2132, Cell Signaling Technology), PINK1 (1:500 dilution; #6946, Cell Signaling Technology), β-actin (1:10,000 dilution; ab8226, Abcam). Incubation with proper peroxidase-conjugated secondary antibody was then performed: goat anti-rabbit IgG antibody (1:1500 dilution; #AP132P, Millipore Corporation) and goat anti-mouse IgG antibody (1:2000 dilution; #12349, Millipore Corporation) in 5% milk-TBST. Protein bands were visualized by chemiluminescence using enhanced chemiluminescence substrate (Millipore Corporation). Images (16 bit grayscale) were acquired using the G:BOXChemi XT4 (Syngene, Cambridge, United Kingdom) system and analyzed using the ImageJ software. Statistical analysis was performed by two-tailed, unpaired Student’s *t*-test and by two-way ANOVA; p < 0.05 was considered significant.

### Shotgun Label-Free Quantitative Proteomics

Whole cell pellets and mitochondrial-enriched fractions were lysed and digested in 0.1% RapiGest SF Surfactant (Waters) solution, as previously described (Alberio et al., 2017). Briefly, after reduction (10 mM TCEP for 30 min at 55°C) and alkylation (20 mM iodoacetamide for 30 min at RT) steps, tryptic digestion was performed overnight at 37°C in RapiGest SF, using a final protease:protein ratio of 1:50 (w/w), with MS-grade trypsin (Promega). Digested peptides were then diluted in a solution of 0.1% formic acid (FA) and 3% acetonitrile (ACN), in order to load 0.25 μg of each digested sample on a 5 μm Symmetry C18 trapping column 180 μm × 20 mm (Waters). Peptides were thus separated by a 120 min reverse phase gradient at 300 nL/min (linear gradient, 2–40% ACN over 90 min) using a HSS T3 C18 1.8 μm, 75 μm × 150 mm nanoscale LC column (Waters Corp.) maintained at 40°C on a UPLC ACQUITY M Class (Waters). Separated peptides were analyzed in a shotgun experiment on a Synapt G2-Si Mass spectrometer (Waters), directly coupled to the chromatographic system. Data have been acquired in High Definition MS^E^ (HDMSE), a data-independent acquisition (DIA) protocol where ion mobility separation (IMS) has been integrated into LC-MS^E^ workflow (Distler et al., 2016).

Mass spectra have been acquired in positive polarity and resolution analyzer mode. TOF MS was operating over 50–2000 m/z using a scan time of 0.5 s and a continuum data format. Data were post-acquisition lock mass corrected using the doubly charged monoisotopic ion of (Glu1)-Fibrinopeptide B (Waters), sampled every 30 s. For IMS, wave height at 40 V, wave velocity of 1.000 m/s and transfer wave velocity of 175 m/s have been applied. Instrument settings were defined to apply a drift time specific transfer collision energy ramp, as described in detail in Supplementary Parameters.

Data from three replicate experiments for each sample were processed for qualitative and quantitative analysis using the ProteinLynx Global Server v. 3.0.3 software (PLGS, Waters). The qualitative identification of proteins was obtained by searching in human database (neXtProt release 2017_08_01). Search parameters were set as: automatic tolerance for precursor ions and for product ions, minimum one fragment ions matched per peptide, minimum three fragment ions matched per protein, minimum one peptide matched per protein, two missed cleavage, carbamidomethylation of cysteines as fixed modification and oxidation of methionines as variable modifications, and false discovery rate (FDR) of the identification algorithm ≤1%.

Tables including quantifications for all identified proteins were generated (Supplementary Tables 1, 2 for mitochondrial and total fractions, respectively). Label free quantitative analysis was obtained by using the protein expression analysis mode integrated in PLGS software, applying an automatic normalization. Filtered tables were generated to include proteins found at least in two out of three technical replicates and to exclude proteins showing less than 30% change (corresponding to a ratio of ±0.38 in log_2_ scale) and those showing no statistical significance according to the PLGS software (Supplementary Tables 3, 4 for mitochondrial and total fractions, respectively). Multiple isoforms were reduced to non-redundant Uniprot IDs and median values for ratio and variance were calculated. *p*-value was calculated from variance, ratio, and sample size using the BSDA package^2^. All data analysis was written using the R environment for statistical computing.

### Systems Biology

The interactions among all significantly altered proteins in *PARK2*-mutated patients (Supplementary Tables 3, 4) were analyzed by STRING^3^. The parameters set for the over-representation analysis were: meaning of network edges = evidence; active interaction sources = experiments, databases, and co-expression; minimum required interaction score = high confidence (0.700). The strictly mitochondrial proteins were identified using the information retrieved by neXtProt^4^ and Gene Ontology Consortium^5^ databases. Only the gold annotations were considered. Eventually, we used the functional mitochondrial human proteome network to visualize the interactions between the strictly mitochondrial proteins and the mitochondrial-associated proteins (Monti et al., 2018). The protein–protein interaction (PPI) networks were visualized using Cytoscape (Shannon et al., 2003). Clusters were built with the ClusterMaker platform and the GLay algorithm (Su et al., 2010). The PPI networks thus generated were used to perform an over-representation analysis using the BiNGO application (Maere et al., 2005).

Gene set enrichment analysis (GSEA) was performed using the ReactomePA package that provides functions for pathway analysis based on Reactome pathways database (Yu and He, 2016). To this purpose, we used the input list generated by including all proteins in controls and *PARK2*-mutated patients both in the mitochondrial (Supplementary Table 1) and in the total fractions (Supplementary Table 2). Proteins that were detected only in either control subjects or in *PARK2*-mutated patients (unique) were excluded. The log_2_(P/C ratio) was used as a metrics for ranking the gene list. Gene IDs were obtained using UniProt.ws, a collection of functions for retrieving, processing, and repackaging the UniProt web services (Carlson and Ortutay, 2019), and the Rentrez library that provides an R interface to the NCBI (Winter, 2017). The parameters set for the analysis were: number of permutations = 10,000; *p*-values cut off = 0.5. *p*-values were adjusted for multiple testing correction using the Benjamini–Hochberg approach, which controls the FDR. Procedures were written using the R environment for statistical computing.

### Generation of the PPI Network and Co-immunoprecipitation

A mitochondrial PPI network was generated by retaining interactions from two or more published studies (PMID are listed in Supplementary Table 5 for each interaction) for proteins whose level was altered in *PARK2*-mutated patients. The network was then visualized using Cytoscape.

A small set of interesting candidate interactions of physiological relevance was confirmed in control subject C4 and *PARK2*-mutated PD patient P3 by immunoprecipitation. Mitochondrial pellets were cross-linked with 1 mM dithiobis (succinimidyl propionate) (DSP; Thermo Fisher Scientific) and lysed with 500 μl RIPA buffer. Inputs were controlled by western blotting. Three microliter of each antibody [HSPA8, abcam (ab51052); HSPD1, abcam (ab46798); ALDH2, Santa Cruz Biotechnology (sc-100496)] were added to 1 mg of mitochondrial protein lysates, which were then incubated for 1 h in agitation at 4°C. One hundred microliter of μMACS magnetic microbeads (Miltenyi) were then added to lysates and incubated with continued agitation ON at 4°C. After washing the microbeads suspensions using 0.1% RIPA buffer through μMACS columns (Miltenyi), proteins were eluted using 200 μl of Laemmli buffer, previously heated at 95°C. Eluates were analyzed by western blotting.

## Results

### Parkin Protein Was Undetectable in Fibroblasts Derived From *PARK2*-Mutated Patients

**FIGURE 1:**
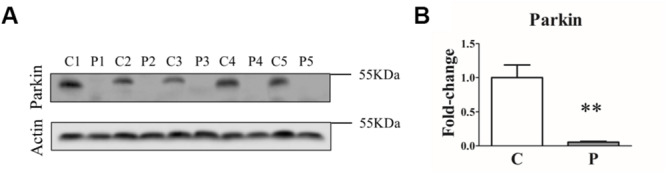
Parkin levels are reduced in *PARK2*-mutated patients. **(A)** Representative western blot image of Parkin protein in primary skin fibroblasts of *PARK2*-mutated patients (P1, P2, P3, P4, and P5) and control subjects (C1, C2, C3, C4, and C5). **(B)** Relative quantification expressed as mean ± SEM (three technical replicates). Data were analyzed by two-tailed, unpaired Student’s *t*-test by comparison of PD patients and control subjects. **p < 0.001.

In order to assess how different genetic mutations actually affected Parkin protein levels, we first analyzed Parkin protein abundance in primary skin fibroblasts from five *PARK2*-mutated patients and five control subjects by western blot. As shown in Figure 1, a complete loss of Parkin protein was observed in all the samples from *PARK2*-mutated patients, independent of the mutation type.

### *PARK2* Mutations Induced a Significant Dissipation of the Mitochondrial Membrane Potential (ΔΨ_m_), Without Causing the Accumulation of PINK1 Protein

**FIGURE 2:**
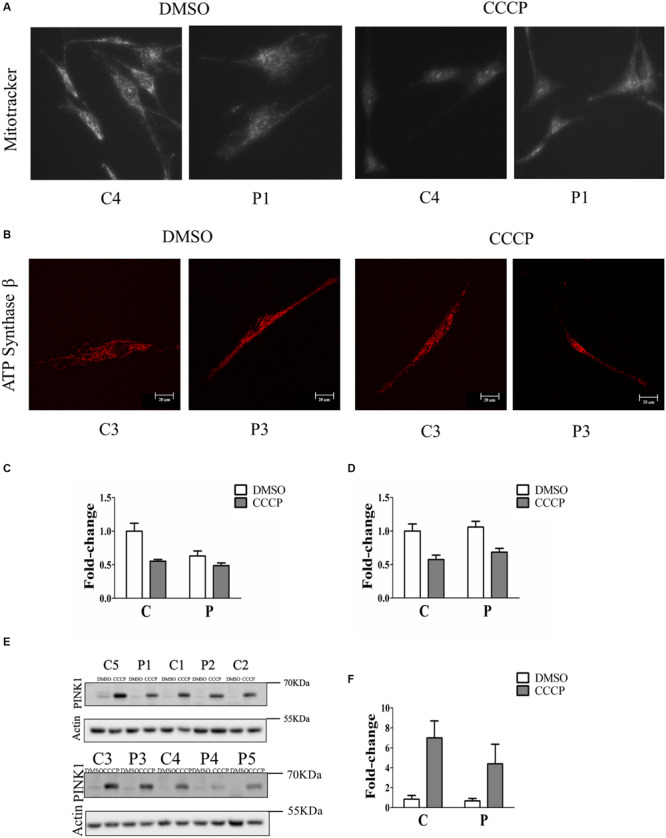
Mitochondrial depolarization without PINK1 protein accumulation in *PARK2*- mutated patients. **(A)** Representative images of fibroblast cells from one control subjects (C4) and one *PARK2*-mutated patient (P1) stained with Mitotracker Red CMXRos after DMSO or CCCP treatment. **(B)** Representative images of fibroblast cells from one control subjects (C3) and one *PARK2*-mutated patient (P3) stained with anti ATP Synthase β after DMSO or CCCP treatment. **(C)** Quantification of Mitotracker Red CMXRos fluorescence normalized by the number of cells. Data, expressed as mean ± SEM, were analyzed by two-way ANOVA, to assess the effect of “mutation,” “treatment,” and “interaction.” “Mutation” (*p* = 0.009; *F* = 9.0) and “treatment” (*p* = 0.001; *F* = 16.2) resulted to be significant sources of variation. **(D)** Quantification of anti ATP Synthase beta fluorescence normalized by cell surface. Data, expressed as mean ± SEM, were analyzed by two-way ANOVA. “Treatment” (*p* = 0.001; *F* = 24.8) resulted to be a significant source of variation. **(E)** Representative western blot image of PINK1 protein after DMSO (control) or CCCP treatment in primary skin fibroblasts of *PARK2*-mutated patients (P) and control subjects (C). **(F)** Relative fold-change of PINK1 protein in both control subjects and *PARK2*-mutated patients after CCCP treatment. Data were expressed as mean ± SEM (three technical replicates). Statistical analysis was performed by two-way ANOVA. “Mutation” (*p* = 0.0001; *F* = 210.9), “treatment” (*p* = 0.0001; *F* = 16.92), and “interaction” (*p* = 0.0007; *F* = 12.75) resulted to be significant sources of variation. “Mutation” = control subjects vs. *PARK2*-mutated patients, “treatment” = DMSO vs. CCCP.

To determine whether the loss of Parkin protein had an impact on mitochondrial function, fibroblasts were stained with Mitotracker Red CMXRos, which accumulates in mitochondria with an intact membrane potential, in the absence and in the presence of the ionophore CCCP. The mitochondrial fluorescence of *PARK2*-mutated fibroblasts was fainter compared to that of control subjects (Figure 2A and Supplementary Figure 2). In order to verify that the observed reduced staining was due to mitochondrial depolarization and not to a reduced number of mitochondria, the ATP Synthase beta signal was quantified by immunofluorescence and normalized by cell surface (Figure 2B). In particular, the mitochondrial membrane potential was reduced to 63% in *PARK2*-mutated patients in the DMSO condition, compared to 55% in control subjects in the CCCP condition and to 49% in *PARK2*-mutated patients in the CCCP condition (Figure 2C). Moreover, the quantitation of the ATP Synthase beta signal by two-way ANOVA revealed that only “treatment” (*p* = 0.001; *F* = 24.8) was a significant source of variation (Figure 2D).

In order to verify whether the mitochondrial depolarization induced the accumulation of PINK1 protein, we evaluated the levels of this protein in whole cell extracts of fibroblasts from control and *PARK2*-mutated patients. Since PINK1 protein was barely detectable at basal conditions, we treated fibroblast cells with CCCP, a protonophore able to positively induce the accumulation of PINK1 and, consequently, the activation of PINK1/Parkin mitophagy pathway in healthy cells (Matsuda et al., 2010), so to highlight possible differences in mitophagy induction. Therefore, fibroblasts were exposed to 60 μm CCCP for 24 h. As shown in Figures 2E,F, CCCP treatment determined the accumulation of PINK1 in control subjects, as expected. On the other hand, PINK1 levels were increased to a lesser extent by CCCP treatment in fibroblast cells from *PARK2*-mutated patients.

### The Mitochondrial Network Morphology Was Not Impaired in *PARK2*-Mutated Patients

**FIGURE 3:**
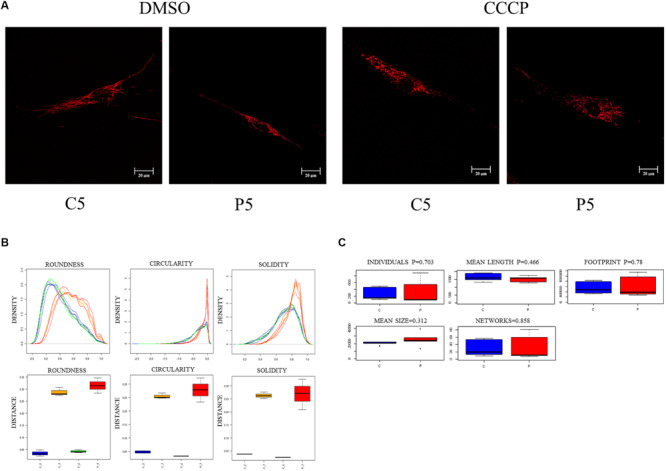
Mitochondrial network morphology is not altered in *PARK2*-mutated samples. **(A)** Representative immunofluorescence images of primary skin fibroblasts from one control subject (C5) and one *PARK2*-mutated patient (P5) stained for ATP synthase β, after DMSO or CCCP treatment. **(B)** Example of three morphological parameters (Circularity after logarithmic transformation, Roundness, and Solidity) analyzed using the “Analyze Particles” tool of ImageJ. Distribution density for all particles analyzed in five fields of view from each subject (blue curves, CTRL subjects/DMSO; orange curves, CTRL subjects/CCCP; green curves, *PARK2*-mutated patients/DMSO; red curves, *PARK2*-mutated patients/CCCP) and Kolmogorov–Smirnov distances of Circularity, Roundness, and Solidity, color codes as above. **(C)** Morphological parameters analyzed using the “Analyze Skeleton” tool. No statistical differences were observed between control subjects and *PARK2*-mutated patients using the univariate non-parametric Wilcoxon test. C, control subjects; P, *PARK2*-mutated patients.

We then investigated the mitochondrial network morphology to verify whether the observed loss of mitochondrial membrane potential in *PARK2*-derived cells could have an impact on mitochondrial dynamics (i.e., fission or fusion). To address this, fibroblast cells were stained with ATP synthase β antibody to visualize the mitochondrial network through confocal microscopy, in the absence and in the presence of CCCP treatment. A branched filamentous mitochondrial network was observed in both *PARK2*-mutated patients and control subjects, with mitochondria distributed all over the cell. As expected, CCCP induced fragmentation of the network in all samples (Figure 3A and Supplementary Figure 3). To give a statistical assessment to this qualitative evaluation, images were analyzed with the “Analyze Particles” function of Image J (Bondi et al., 2016). By employing this strategy, it was possible to measure several morphological parameters that describe the mitochondrial network shape. Among the parameters, the distribution density of Circularity, Roundness, and Solidity are shown in Figure 3B. All other parameters are reported in Supplementary Figure 4. For listed parameters, two-way ANOVA of distances showed that treatment was a significant source of variance (*p* = 0.0001, 1.3 × 10^–8^ and 4.2 × 10^–5^, respectively). Mutation and Interaction did not contribute significantly.

To further describe the mitochondrial network morphology, we decided to use the “Analyze Skeleton” function of Image J (Valente et al., 2017). This tool gives additional information about the shape of the network, in particular regarding junctions and branches. Five parameters (i.e., individuals, mean length, mean size, footprint, and networks) were compared between *PARK2*-mutated patients and control subjects (Figure 3C). Also this analysis did not show any significant difference in the evaluated parameters, using the univariate non-parametric Wilcoxon test. These results demonstrated that the mitochondrial network morphology was not perturbed (i.e., fission or hyper-fusion processes) despite the mitochondrial depolarization observed in *PARK2*-mutated fibroblasts. Given the non-branched characteristics of mitochondrial networks in CCCP-treated cells, this analysis was limited to untreated cells.

**FIGURE 4:**
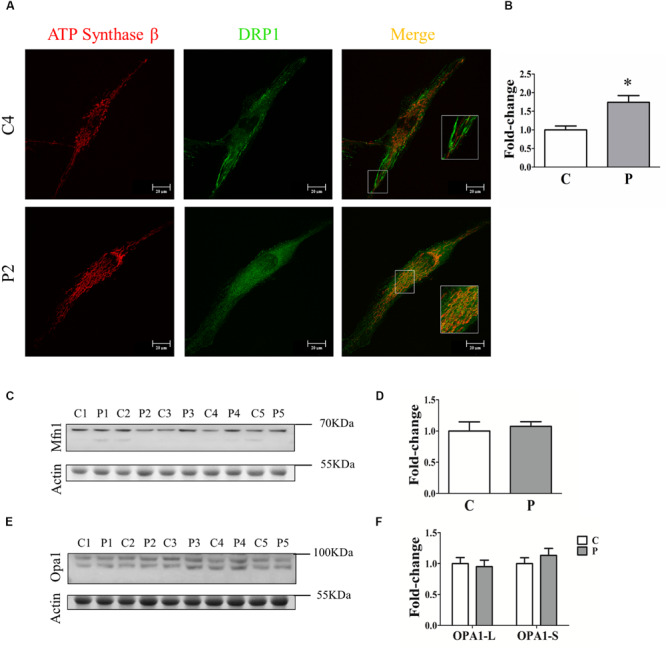
Quantification of fission and fusion proteins. **(A)** Representative images of ATP synthase β (mitochondrial area) and DRP1 (quantification) signals from one control subject (C4) and one *PARK2*-mutated patient (P2). The merge panel shows the juxtaposition of the two signals. Insets show the magnification (165%) of details in squares. **(B)** Quantification of the DRP1 signal per mitochondrial surface unit. Data were expressed as mean ± SEM. ^∗^*p* = 0.026. **(C)** Representative western blot image of MFN1 protein in primary skin fibroblasts of *PARK2*-mutated patients (P1, P2, P3, P4, and P5) and control subjects (C1, C2, C3, C4, and C5). **(D)** Relative quantification expressed as mean ± SEM. Data were analyzed by two-tailed, unpaired Student’s *t*-test by comparison of PD patients and control subjects. **(E)** Representative western blot image of OPA1 protein in primary skin fibroblasts of *PARK2*-mutated patients (P1, P2, P3, P4, and P5) and control subjects (C1, C2, C3, C4, and C5). **(F)** Relative quantification expressed as mean ± SEM. Data were analyzed by two-tailed, unpaired Student’s *t*-test by comparison of PD patients and control subjects.

To clarify the absence of mitochondrial network morphology alterations in *PARK2*-mutated patients, we investigated the recruitment of DRP1 to mitochondria to explore the fission process. To this end, quantification analysis of DRP1 was performed in the area defined by ATP synthase β, a mitochondrial marker (Figure 4A). Surprisingly, we found that DRP1 recruitment to mitochondria was significantly increased by 74% in PARK2-mutated patients, suggesting that DRP1 recruitment is Parkin-independent (Figure 4B). To better explore the fusion process, the abundance of two different proteins involved in mitochondrial fusion, i.e., mitofusin 1 (MFN1) and optic atrophy 1 (OPA1), were measured by western blot analysis (Figures 4C,E). As shown in Figures 4D,F, MFN1 and OPA1 (both the long OPA1-L and the short form OPA1-S) levels were not altered, suggesting that the fusion process is not blocked.

### Mitochondrial and Whole-Cell Proteomes Were Altered by *PARK2* Mutations

**FIGURE 5:**
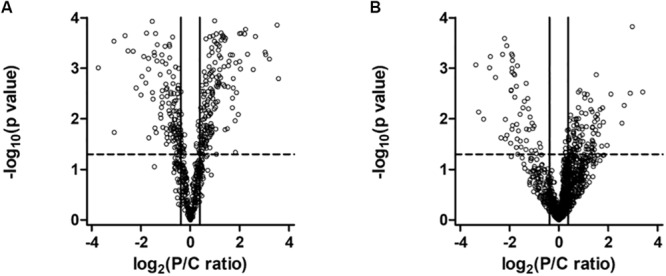
Volcano plot of proteins in the mitochondrial **(A)** and in the whole cell **(B)** fractions. Dots represent proteins, distributed by fold change along the *x*-axis and by *p*-value along the *y*-axis. Proteins with a statistically significant differential expression are located in the top right (up-regulated) and top left (down-regulated) quadrants.

To understand the effect of *PARK2* mutations on the mitochondria and total proteome, we performed a label-free quantitative proteomic analysis on enriched mitochondrial and whole cell protein fractions. Quantitative data were graphically represented with a Volcano plot (Figure 5), showing the selected thresholds for statistical significance and fold-change. A comparable number of up- and down-regulated proteins between the two conditions, both in the mitochondria (Figure 5A) and whole cell fractions (Figure 5B), indicated the good quality of the shotgun analysis. Extended information on the proteins used to generate the Volcano plots can be found in Supplementary Table 1 (649 proteins in the mitochondrial fraction) and Supplementary Table 2 (1457 in the total fraction). Proteins whose level was significantly altered in *PARK2*-mutated samples were 227 in the mitochondrial fraction (Supplementary Table 3) and 168 in the total fraction (Supplementary Table 4), indicating the central role played by Parkin at the mitochondrial level.

### Several Molecular Pathways Were Altered by Mutations in *PARK2* Gene

**FIGURE 6:**
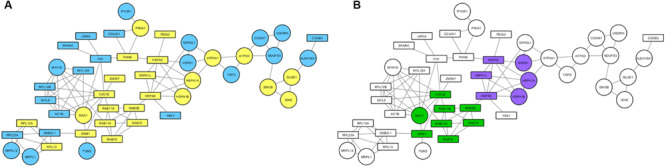
Mitochondrial pathways altered in *PARK2*-mutated samples. PPI network was built considering both strictly mitochondrial and mitochondria-associated proteins. **(A)** Round nodes, strictly mitochondrial proteins; rectangular nodes, mitochondria-associated proteins; yellow nodes, up-regulated in *PARK2*-mutated patients; blue nodes, down-regulated in *PARK2*-mutated patients. **(B)** Over-represented pathways mapped on the PPI network. Green nodes, small GTPases mediated signal transduction pathway; purple nodes, unfolded protein response pathway.

Proteins found to be altered by *PARK2* mutations in the mitochondrial fraction were subjected to a network-based analysis, using STRING database as reference, to highlight molecular pathways altered by Parkin loss. First, the mitochondrial input list (Supplementary Table 3; 227 proteins up- or down-regulated or unique to one category) was filtered by considering only proteins present in the functional mitochondrial network (Monti et al., 2018), which includes both mitochondrial and mitochondria-associated proteins in the neXtProt and/or the Gene Ontology databases. Eighty-three proteins were mapped on this reference network. This list was then used to build a protein network, retrieving edge information from the STRING database, thus obtaining a PPI cluster made by 48 protein nodes (Figure 6A). The over-representation analysis using BiNGO revealed that the unfolded protein response (purple nodes) and the small GTPases mediated signal transduction (green nodes) were altered by *PARK2* mutations (Figure 6B).

**FIGURE 7:**
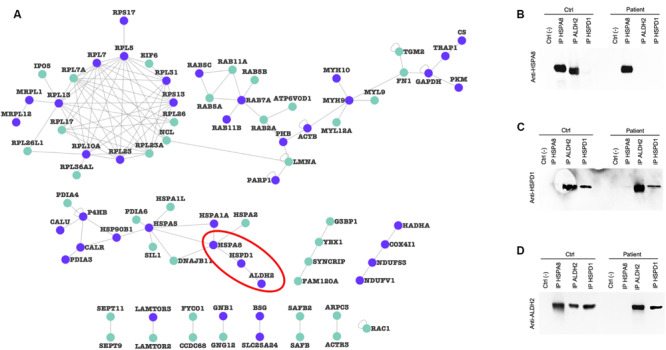
Protein-protein interaction alterations. **(A)** PPI-network of altered proteins in the mitochondrial fraction. Purple nodes, strictly mitochondrial proteins; light green nodes, mitochondria-associated proteins. **(B–D)** Co-IP experiments of indicated prey proteins (HSPA8, HSPD1, and ALDH2, red line in panel **(A)** in mitochondrial-enriched fractions obtained from fibroblasts of C4 control subject and P3 *PARK2*-mutated subject using specific antibodies. Protein G beads served as a negative control. Blots were probed using HSPA8 **(B)**, HSPD1 **(C)**, and ALDH2 **(D)** antibodies.

To verify whether alterations in protein levels affected mitochondrial proteins organized in macromolecular complexes, we filtered the mitochondrial input list (Supplementary Table 3), searching for protein pairs that have been shown to interact in at least two publications (Supplementary Table 5) to generate a validated PPI network. Figure 7A shows the resulting mitochondrial interactome, characterized by purple nodes (strictly mitochondrial proteins) and cyan nodes (mitochondria-associated proteins). We identified a mitochondrial cluster (highlighted with the red line) composed of three mitochondrial proteins (HSPA8, HSPD1, ALDH2). We then decided to verify if the altered expression of these proteins in *PARK2*-mutated samples could alter the PPI composition of this complex. As a result, we found that the binding between HSPA8 and ALDH2 is lost in a *PARK2* patient when compared to the control subject (Figure 7B). On the other hand, the interaction between ALDH2 and HSPD1 is not impaired by *PARK2* mutation (Figures 7C,D and Supplementary Figure 5).

**FIGURE 8:**
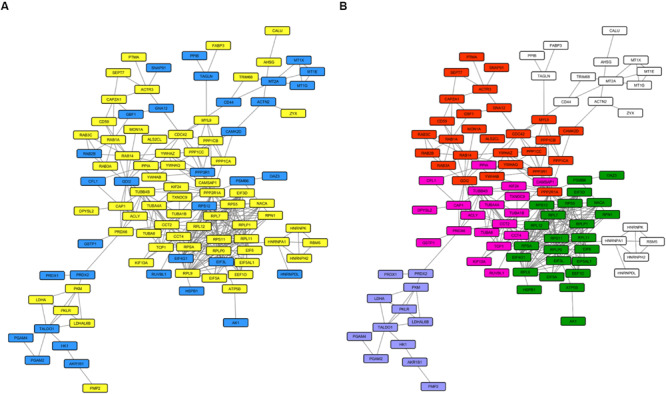
Molecular pathways altered in the whole cell proteome of *PARK2*-mutated patients. The PPI network includes 100 nodes. **(A)** Yellow nodes, up-regulated proteins in *PARK2*-mutated patients; blue nodes, down-regulated in *PARK2*-mutated patients. **(B)** Over-represented pathways mapped on the PPI network. Pink nodes, microtubule-based movement; green nodes, translation; red nodes, signal transmission and transduction; purple nodes, glucose and hexose catabolic pathway.

We then investigated the whole-cell proteome alterations. First of all, we created an input list from the 168 proteins that changed in *PARK2*-mutated patients. This input list (Supplementary Table 4) was used to generate a PPI network including 100 proteins (Figure 8A), using the STRING database as reference. The network was divided in sub-clusters by the GLay algorithm and each cluster was analyzed by over-representation analysis using BiNGO. This resulted in the identification of four molecular pathways that were mainly altered by *PARK2* mutations, i.e., signal transmission and transduction, microtubule-based movement, translation, and glucose and hexose catabolic pathways (Figure 8B).

**FIGURE 9:**
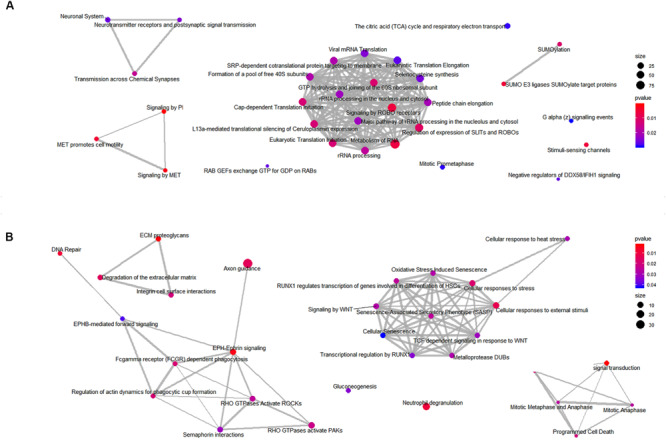
Enriched classes of proteins found using a GSEA approach. Enriched map of GSEA results for the mitochondrial **(A)** and the total **(B)** fractions. Color coding represents significance of the gene set for the dataset. Node size correspond to the number of genes from the reference set that are part of the gene set.

Next, to identify the over-represented classes of proteins, we performed GSEA on the mitochondrial and whole cells’ fractions. To carry out this analysis, 649 and 1457 proteins were considered for the mitochondrial and the total fraction, respectively (Supplementary Tables 1, 2). To visualize the results, we plotted the enriched pathways as graphs, identifying several significantly enriched pathways. In particular, the analysis revealed an enrichment of processes related to the RHO GTPases and the axon guidance for the mitochondria fraction (Figure 9A) and the Rab GEFs exchange GTP for GDP on Rabs for the total fraction (Figure 9B). All identified enriched pathways both in the mitochondrial and in the total fractions are shown in Supplementary Tables 6, 7.

### Rab7A Was Recruited to Mitochondria in PARK2 Patients

**FIGURE 10:**
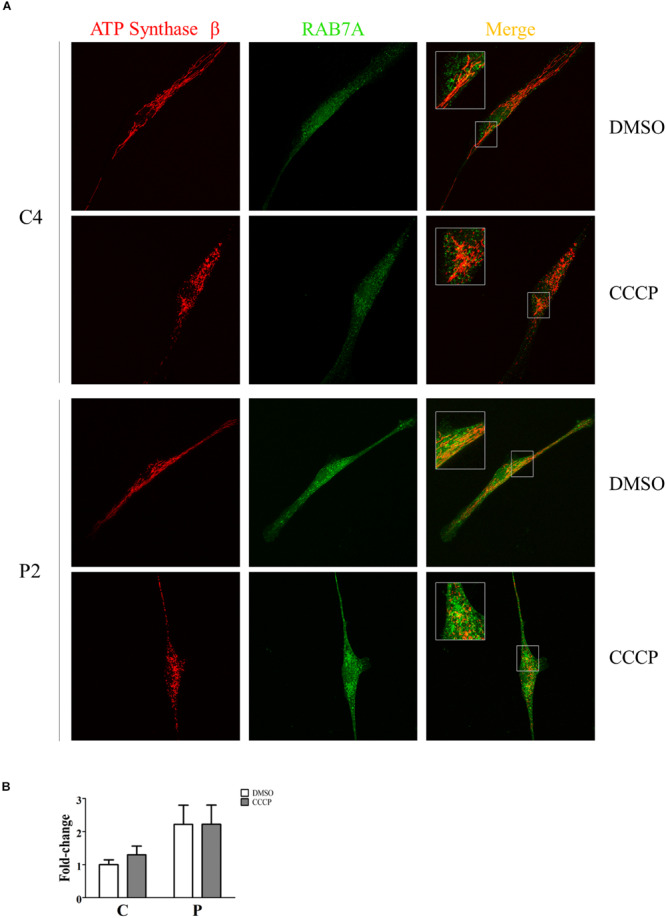
Rab7A was recruited to mitochondria in PARK2 patients. **(A)** Representative images of ATP synthase β (mitochondrial area) and Rab7A (quantification) signals from one control subject (C4) and one *PARK2*-mutated patient (P2) after DMSO or CCCP treatment. The merge panel shows the juxtaposition of the two signals. Insets show the magnification (165%) of details in squares. **(B)** Quantification of Rab7A signal per mitochondrial surface unit. Data were expressed as mean ± SEM. Statistical analysis was performed by two-way ANOVA, to assess the effect of “mutation” (control subjects vs. PARK2 patients), “treatment” (DMSO vs. CCCP), and “interaction.” “Treatment” (*p* = 0.74; *F* = 0.12) and “interaction” (*p* = 0.74; *F* = 0.11) did not result to be significant sources of variation. “Mutation” (*p* = 0.04; *F* = 5.97) resulted to be a significant source of variation.

The recruitment of Rab7A to mitochondria was investigated by immunofluorescence. To this end, quantification analysis of Rab7A protein was performed in the ATP Synthase β area in the absence and in the presence of CCCP. As shown in Figure 10, we observed a significant two-fold increase of Rab7A localization at the mitochondrial level in *PARK2* patients (*p* = 0.04), thus validating our proteomics finding in the mitochondrial fractions. On the other hand, the trigger of the mitophagy process by CCCP did not amplify the observed recruitment.

## Discussion

Parkinson’s disease is a complex and multifactorial disorder whose etiology has not been clarified yet. Nevertheless, it is well-known that many of the molecular pathways implicated in PD etiology converge on mitochondria, resulting in their dysfunction. In this pathological landscape, mitophagy impairment and the consequent accumulation of damaged mitochondria seems to play a pivotal role in PD onset and progression (Fernández-Moriano et al., 2015; Zilocchi et al., 2018). Since *PARK2*-mutated patients offer a unique pathological background in which PD pathology is driven by Parkin loss-of-function and PINK1/Parkin mitophagy impairment (Youle and Narendra, 2011), we decided to verify the whole-cell and mitochondrial quantitative proteomic alterations that characterize these PD patients. To have a more comprehensive view of the mitochondrial alterations due to *PARK2* mutations, we also investigated the impact of Parkin loss-of-function on mitochondrial polarization and network morphology.

To achieve these, we used primary skin fibroblasts obtained from five *PARK2*-mutated PD patients, which were all characterized by the absence of Parkin protein, independent of the type of genetic mutation that affected these subjects. Fibroblasts are an easily accessible source of proliferating cells that can be sub-cultured for long periods using basic equipment, thus giving the possibility to study the disease and its pathological mechanisms in several fibroblast cell lines. Fibroblast cellular model is widely used in the neurodegenerative disease research field given its ability to recapitulate the polygenic risk factor, the cell damage at the age of the patient, and some of the biochemical alterations that characterize neurons affected by the disease (Auburger et al., 2012; Kilpatrick et al., 2016). The use of this cellular model is further supported by the numerous studies that characterized mitochondrial dysfunctions in both sporadic and genetic forms of PD. Indeed, fibroblasts obtained from the skin biopsies of *PARK2*-mutated patients show alterations in mitochondrial bioenergetics (lower membrane potential, complex I activity and ATP cellular levels) (Zanellati et al., 2015). Despite all these features, the main concern is that fibroblasts do not represent the tissue directly affected by the disease. Furthermore, there is the obvious limitation of using cells that do not strictly rely on mitochondria for ATP production. However, fibroblasts constitute a unique opportunity to investigate the molecular effects of Parkin loss in several primary cell lines, with different genetic, epigenetic, and environmental backgrounds.

To have a more comprehensive view of multifactorial and complex disorders, we decided to use a label free quantitative proteomic approach to investigate the mitochondrial and the total proteome alterations that characterize *PARK2* pathology. Indeed, this approach allowed us the detection and quantification of several hundreds of proteins, thus highlighting the protein expression level difference between *PARK2*-mutated patients and control subjects. The quantitative proteomic analysis performed on the mitochondria-enriched fractions showed 227 proteins whose levels were altered by *PARK2* mutations. On the other hand, the quantitative proteomic analysis performed on the whole cell fractions showed 168 proteins whose levels were altered by *PARK2* mutations. These results revealed that 35% of mitochondrial proteins identified in our study quantitatively changed in *PARK2*-mutated patients, compared to 11% proteins in whole cell extracts, thus highlighting a critical impact of Parkin loss-of-function on the mitochondrial proteome.

To elucidate the molecular pathways altered in this familial form of PD, we performed a network-based analysis in order to visualize interactions among the 227 mitochondrial proteins differentially expressed. We performed an over-representation analysis (Fasano et al., 2016) on the resulting PPI network, thus revealing several biochemical pathways altered by *PARK2* mutations. In particular, the use of this approach helped us to identify the unfolded protein response and small GTPases mediated signal transduction as the two main pathways altered by the disease. Indeed, the expression levels of several heat shock proteins (HSPs) were affected. HSPs consist of a heterogeneous group of highly conserved proteins that are fundamental in maintaining cellular homeostasis. Moreover, Parkin and HSP70 pathways seem to be interconnected, since Parkin is able to ubiquitinate HSP70 at several sites (Moore et al., 2008) and a tight association between chaperone systems and PD pathology has been demonstrated (Kalia et al., 2004).

The second mitochondrial pathway altered by *PARK2* mutations was the small GTPases, mediating signal transduction. The role of Rab proteins in PD pathology has recently been proposed. The discovery of familial PD cases caused by mutations in *RAB39B* and *RAB32* (Gao et al., 2018) pointed out a new cellular pathway impairment that characterize this pathology. Indeed, Rab proteins are essential for the spatiotemporal localization of proteins, which, in turn, determines their intracellular functions and/or disposal (Gao et al., 2018). Recently, a tight relation between Rabs and mitophagy has been discovered (Yamano et al., 2014; Hammerling et al., 2017), suggesting that Parkin acts in the removal of damaged mitochondria not only by the canonical PINK1/Parkin pathway but also interacting with the Rab endosomal pathway. The quantitative alteration of Rab proteins at the mitochondrial level, demonstrated by our proteomics data, strongly support the importance of these proteins in mitochondrial homeostasis and their involvement in PD pathology. The role of Rab alteration in PD pathology was further highlighted by the over-representation and the GSEA conducted on the proteins significantly altered by *PARK2* mutation in the total fractions.

Given the fundamental role of Rab proteins in the mitophagy process and the re-localization to mitochondria of Rab7A for the mitophagosome formation (Yamano et al., 2018; Tan and Tang, 2019), we investigated the mitochondrial localization of Rab7A by immunofluorescence. As a result, we verified the re-localization at mitochondria of Rab7A in *PARK2* patients, whereas no significant variations were observed to be associated with CCCP treatment, nor with the interaction between the two factors. This observation may indicate that in *PARK2* patients derived fibroblasts Rab7A is recruited to mitochondria because of their depolarization. However, the mitophagic process is not completed.

In general, mitochondrial dynamics seem to be partially altered by Parkin loss. First, we assessed the mitochondrial membrane potential using Mitotracker Red CMXRos. As a result, we observed a reduction of the mitochondrial membrane potential in *PARK2*-mutated patients, as demonstrated by previous studies (Grünewald et al., 2010; Zanellati et al., 2015), to an extent similar to that induced by CCCP treatment. We then decided to verify whether the mitochondrial depolarization, the main trigger for the activation of the PINK1/Parkin mitophagy pathway, could induce the accumulation of PINK1 protein (Matsuda et al., 2010; Narendra et al., 2010). Despite the lower mitochondrial polarization, PINK1 was undetectable both in fibroblasts derived from control subjects and *PARK2*-mutated patients. Since several studies used the protonophore CCCP as an established cellular model to induce the PINK1/Parkin mitophagy (Gegg et al., 2010; Rakovic et al., 2011; Gao et al., 2015; Bondi et al., 2016), we decided to assess the accumulation of PINK1 after inducing the activation of this mitochondrial disposal pathway. As expected, CCCP treatment determined the accumulation of PINK1 in control subjects. On the other hand, PINK1 levels were drastically reduced in skin fibroblasts from *PARK2*-mutated patients exposed to CCCP, thus suggesting that Parkin protein is necessary for the accumulation of PINK1 on the mitochondrial surface and that mitochondrial depolarization is not the only process that triggers the activation of mitophagy.

To better describe the mitochondrial alterations that occur in our fibroblast model, we performed an analysis of the mitochondrial network morphology. We analyzed the distribution density of several morphological parameters, using both the “Analyze Particles” and “Analyze Skeleton” tools of ImageJ (Bondi et al., 2016; Valente et al., 2017). We did not observe any significant difference in all the evaluated parameters between *PARK2*-mutated patients and control subjects, whereas CCCP is able to disrupt the mitochondrial network in all samples. This result is in agreement with the filamentous mitochondrial network visible in our confocal images and with previously published studies (Grünewald et al., 2010; Zanellati et al., 2015) and suggests that *PARK2* loss-of-function does not affect the morphology of the mitochondrial network. To find a molecular correlate to these observations, we verified OPA1 and MFN1 levels and DRP1 mitochondrial localization in *PARK2*-mutated patients. While DRP1 localizes to depolarized mitochondria, OPA1 and MFN1 levels are not altered. As a whole, it seems that even if fission is triggered (DRP1 accumulation), fusion is not blocked (lack of OPA1 and MFN1 elimination). If we consider also the Rab7A mitochondrial localization, we can conclude that some molecular pathways are activated by mitochondrial depolarization in *PARK2*-mutated patients. Nevertheless, mitophagy is not properly completed. Further studies are needed to mechanistically describe these impairments in mitochondrial dynamics.

The quantitative proteomic investigation performed on the whole-cell lysates, coupled with a systems biology approach, allowed us to identify other molecular pathways altered by Parkin loss, i.e., signal transmission and transduction, microtubule-based movement, translation, and glucose and hexose catabolic pathways. This analysis depicted a global impairment that affect cellular homeostasis at different level.

To verify whether the quantitative mitochondrial proteome alteration observed in these patients could affect also the composition of protein complexes, we decided to perform a co-immunoprecipitation experiment on the mitochondrial fractions obtained from one control and one *PARK2*-mutated subjects. After building a PPI network using interactomics data from at least two published studies, we identified a small cluster composed by three strictly mitochondrial proteins (HSPA8, HSPD1, and ALDH2). These interactions were chosen since the unfolded protein response pathway was altered in our over-representation analysis. As a result, we found that the binding between HSPA8 and ALDH2 is lost in a *PARK2* patient when compared to the control subject, suggesting that the altered protein expression may have resulted in the alteration of this protein complex binding. This data shows that the mutation in Parkin alters protein interactions. However, further investigation of the interactome impairment is necessary to better understand the pathological landscape caused by Parkin loss-of-function. Moreover, it would be important to repeat key experiments in fibroblasts KO for *PARK2* or in *PARK2*-derived fibroblasts with Parkin re-expressed at physiological levels to confirm changes observed.

## Conclusion

In conclusion, our study demonstrated that Parkin loss-of-function determines the alteration of several biochemical pathways at both mitochondrial and whole-cell levels. In particular, we highlighted the impairment of the Rab pathway using a quantitative proteomic approach, thus emphasizing the tight link between Parkin and Rab dynamics in mitochondrial homeostasis. These quantitative proteome alterations can also influence the interactome, as we demonstrated with a co-immunoprecipitation experiment conducted on a mitochondrial protein complex. Moreover, the mitochondrial depolarization, the lack of PINK1 accumulation (even when triggered with CCCP), and the absence of fission process in *PARK2*-mutated patients depict a general mitochondrial impairment that affect these organelles at different levels.

## Data Availability Statement

The datasets generated for this study can be found in the ProteomeXchange (Accession: PXD015880).

## Ethics Statement

The studies involving human participants were reviewed and approved by “Cell line and DNA Bank of Genetic Movement Disorders and Mitochondrial Diseases” of the Telethon Network of Genetic Biobanks (TNGB). The patients/participants provided their written informed consent to participate in this study.

## Author Contributions

TA, BG, LP, and MB designed the experiments. IC, ML, MM, and MZ carried out the biochemistry and cellular biology experiments. VC, FM, and LP carried out the LC-MS/MS analysis. IC, MF, MZ, and TA carried out the systems biology analysis. MZ, MB, MTM, and SP carried out the interactomics analysis. IC and MZ wrote the manuscript. TA, MF, and ML revised the manuscript. All authors read and approved the final version of the manuscript.

## Conflict of Interest

The authors declare that the research was conducted in the absence of any commercial or financial relationships that could be construed as a potential conflict of interest.

## Footnotes

1 http://www.r-project.org/

2 https://CRAN.R-project.org/package=BSDA

3 https://string-db.org

4 https://www.nextprot.org

5 http://geneontology.org
