# Supplementary material for: Exploring the Impact of PARK2 Mutations on the Total and Mitochondrial Proteome of Human Skin Fibroblasts
Source: Front Cell Dev Biol. 2020 Jun 11;8:423. doi: 10.3389/fcell.2020.00423 (PMC7300190; doi:10.3389/fcell.2020.00423)
Supplement: Supplementary file 8 [file Data_Sheet_1.PDF]

SUPPLEMENTARY PARAMETERS

Instruments Parameters for C:\Projects\July2015.PRO\ACQUDB\LP_HDMSe_125.EXP
Created by 4.1 SCN932

Lock Spray Configuration:

Reference Scan Frequency(sec)	60.000
Reference Cone Voltage(V)	40.000
Reference Trap Collision Energy	4.000
Reference DRE Setting	99.900

Temperature Correction:

Temperature Correction	Disabled

Instrument Configuration:

Lteff	1800.0
Veff	7197.16
Resolution	20000
Min Points in Peak	2
Acquisition Device	WatersADC
Acquisition Algorithm	ADC Mode
ADC Trigger Threshold (V)	1.00
ADC Input Offset (V)	-1.65
Average Single Ion Intensity	23
ADC Amplitude Threshold	3
ADC Centroid Threshold	-1
ADC Ion Area Threshold	3
ADC Ion Area Offset	10
ADC Pushes Per IMS Increment	1
EDC Delay Coefficient	1.3400
EDC Delay Offset	0.4000

Experimental Instrument Parameters
Instrument Parameter Filename

Polarity	ES+
Capillary (kV)	3.0000
Source Temperature (°C)	80
Sampling Cone	60.0000
Source Offset	80.0000
Source Gas Flow (mL/min)	0.00

Desolvation Temperature (°C)	150
Cone Gas Flow (L/Hr)	30.0
Nanoflow Gas Pressure (Bar)	0.3
Purge Gas Flow (mL/h)	600.0
Desolvation Gas Flow (L/Hr)	600.0
Nebuliser Gas Flow (Bar)	6.5
LM Resolution	4.7
HM Resolution	15.0
Aperture 1	0.0
Pre-filter	2.0
Ion Energy	0.5
Manual Trap Collision Energy	FALSE
Trap Collision Energy	4.0
Manual Transfer Collision Energy	FALSE
Transfer Collision Energy	2.0
Manual Gas Control	FALSE
Trap Gas Flow (mL/min)	2.00
HeliumCellGasFlow	180.00
IMS Gas Flow (mL/min)	90.00
Detector	3150
DetectorCache	2150
Sample Infusion Flow Rate (µL/min)	2
Sample Flow State	Infusion
Sample Fill Volume (µL)	250
Sample Reservoir	A
LockSpray Infusion Flow Rate (µL/min)	1
LockSpray Flow State	Infusion
LockSpray Reservoir	A
LockSpray Capillary (kV)	3.0
Use Manual LockSpray Collision Energy	FALSE
Collision Energy	4.0
Acceleration1	55.0
Acceleration2	200.0
Aperture2	70.0
Transport1	70.0
Transport2	70.0
Steering	-0.40
Tube Lens	75
Pusher	1900.0
Pusher Offset	-0.42
Puller	1370.0
Pusher Cycle Time (µs)	Automatic

Pusher Width (µs)	Automatic
Collector	50
Collector Pulse	10.0
Stopper	10
Stopper Pulse	20.0
Entrance	62
Static Offset	180
Puller Offset	0.00
Reflectron Grid (kV)	1.473
Flight Tube (kV)	10.00
Reflectron (kV)	3.780
Use Manual Trap DC	FALSE
Trap DC Entrance	0.0
Trap DC Bias	45.0
Trap DC	0.0
Trap DC Exit	3.0
Use Manual IMS DC	FALSE
IMS DC Entrance	20.0
Helium Cell DC	50.0
Helium Exit	-20.0
IMSBias	3.0
IMS DC Exit	0.0
USe Manual Transfer DC	FALSE
Transfer DC Entrance	4.0
Transfer DC Exit	15.0
Trap Manual Control	OFF
Trap Wave Velocity (m/s)	311
Trap Wave Height (V)	4.0
IMS Manual Control	OFF
IMS Wave Velocity (m/s)	650
IMS Wave Height (V)	40.0
Transfer Manual Control	OFF
Transfer Wave Velocity (m/s)	175
Transfer Wave Height (V)	4.0
Step Wave 1 In Manual Control	OFF
Enable Reverse Operation	OFF
Step Wave 1 In Velocity (m/s)	300.0
Step Wave 1 In Height	10.0
Step Wave 1 Out Manual Control	OFF
Step Wave 1 Out Velocity (m/s)	300.0
Step Wave 1 Out Height	0.0
Step Wave 2 Manual Control	OFF

Step Wave 2 Velocity (m/s)	300.0
Step Wave 2 Height	0.0
Use Manual Step Wave DC	OFF
Step Wave TransferOffset	25.0
Step Wave DiffAperture1	3.0
Step Wave DiffAperture2	0.0
Use Automatic RF Settings	TRUE
StepWave1RFOffset	300.0
StepWave2RFOffset	350.0
Target Enhancement Enabled	FALSE
Target Enhancement Mode	EDC
Target Enhancement Mass	556.0
Target Enhancement Trap Height (V)	12.0
Target Enhancement Extract Height (V)	8.0
Mobility Trapping Manual Release Enabled	FALSE
Mobility Trapping Release Time (µs)	500
Mobility Trap Height (V)	15.0
Mobility Extract Height (V)	0.0
Trag Gate LUT table enabled	FALSE
TriWave Trap Gate LookUp Table	
Using Drift Time Trimming	FALSE
Drift Time Bins	0
Using Mobility Delay after Trap Release	TRUE
IMS Wave Delay (µs)	1000
Variable Wave Height Enabled	FALSE
Wave Height Ramp Type	Linear
Wave Height Start (V)	10.0
Wave Height End (V)	40.0
Wave Height Using Full IMS	TRUE
Wave Height Ramp (%)	100.0
Wave Height Look Up Table	
Variable Wave Velocity Enabled	FALSE
Wave Velocity Ramp Type	Linear

Wave Velocity Start (m/s)	1000.0
Wave Velocity End (m/s)	300.0
Wave Velocity Using Full IMS	TRUE
Wave Velocity Ramp (%)	100.0
Wave Velocity Look Up Table	
Backing	3.61e0
Source	8.72e-3
Sample Plate	1.00e-6
Trap	2.90e-2
Helium Cell	3.98e0
IMS	2.87e0
Transfer	2.69e-2
TOF	9.68e-7
IMSRFOffset	300
IMSMobilityRFOffset	250
TrapRFOffset	300
Use Automatic RF Settings	TRUE
AutoStepWave1RFOffset	300
AutoStepWave2RFOffset	350
TransferRFOffset	350
MS Profile Type	Profile
MSProfileMass1	300
MSProfileDwellTime1	25
MSProfileRampTime1	25
MSProfileMass2	400
MSProfileDwellTime2	25
MSProfileRampTime2	25
MSProfileMass3	500
PusherInterval	69.000000
PusherOffset	0.250000
LockMassValidSigma	5

Acquisition mass range

Start mass	50.000
End mass	2000.000

Calibration mass range

Start mass	72.140
End mass	1285.325

Experiment Reference Compound Name: GFP_RES_SP_MS

Function Parameters - Function 1 - TOF PARENT FUNCTION

[ACQUISITION]	
Survey Start Time	5.0
Survey End Time	125.0
Survey Ion Mode	ES Mode
Survey Polarity	Positive
[PARENT MS SURVEY]	
Survey Start Mass	50.0
Survey End Mass	2000.0
Parent Survey Low CE (V)	10.0
TIC Threshold	5.0
Survey Scan Time	0.5
Survey Interscan Time	0.0
Survey Data Format	Continuum
Analyser	Resolution Mode
ADC Sample Frequency (GHz)	3.0
TargetEnhancementMass2	69.0
TargetEnhancementMass3	1.75
Survey Use Tune Page CV	NO
Cone (V)	35.0
[PRODUCT IONS]	
Use High CE Product Ions Mass List File High CE Product Ions Mass List Filename	NO
Product Ions Match Logic	NO
Product Ions Switch Threshold (Intensity/s)	10.0
Product Ions Switch Detection Window +/- (mDa)	100.0
Product Ions Retention Time Window +/- (sec)	10.0
[NEUTRAL LOSS]	
Use Neutral Loss Mass List File	NO
Neutral Loss Mass List Filename	
Neutral Loss Match Logic	OR
Neutral Loss Switch Threshold (Intensity/s)	10.0
Neutral Loss Switch Detection Window +/- (mDa)	100.0
[MS/MS]	
MSMS Start Mass	50.0
MSMS End Mass	2000.0
Number of components	0
Use MSMS to MS Switch After Time	NO

MSMS Switch After Time (sec)	10.0
Absence of Neutral Loss	NO
Absence of Product Ion	NO
MSMS Scan Time (sec)	1.0
MSMS Interscan Time (sec)	0.0
MSMS Data Format	Continuum
Use Tune Page Cone Voltage	YES
Use MS/MS ipr File	NO
Instrument Parameter Filename [PEAK DETECTION]	
Peak Detection Window	1.0
Use Intensity based Peak Detection	YES
Charge State Tolerance Window	0.2
Charge State Extraction Window	4.0
Deisotope Tolerance Window	0.2
Deisotope Extraction Window	4.0
Discard survey data [COLLISION ENERGY]	NO
Using Auto Trap MS Collision Energy (eV)	4.000000
Using Auto Transfer MS Collision Energy (eV) [INCLUDE]	2.000000
Precursor Selection [EXCLUDE]	Everything
Use Exclude Masses List	NO
Exclude Mass Range	
Use Exclude File Masses Exclude Mass Filename	NO
Exclude Window +/- (mDa)	100.0
Exclude Retention Time Window [MOBILITY]	10.0
Use Precursor Drift Times	NO
Maintain Mobility Separation	YES
Override IMS Wave Velocity	YES
Override Transfer Wave Velocity	YES
IMS Wave Velocity	1000.0
Transfer Wave Velocity	175.0
Reference Centroid Average	
Reference Frequency	0.0
Reference Cone Voltage	0.0
Calibration	Dynamic 2

Function Parameters - Function 2 - TOF PARENT FUNCTION [ACQUISITION]

Survey Start Time	5.0
Survey End Time	125.0

Survey Ion Mode	ES Mode
Survey Polarity	Positive
[PARENT MS SURVEY]	
Survey Start Mass	50.0
Survey End Mass	2000.0
Parent Survey High CE (V)	30.0
TIC Threshold	5.0
Survey Scan Time	0.5
Survey Interscan Time	0.0
Survey Data Format	Continuum
Analyser	Resolution Mode
ADC Sample Frequency (GHz)	3.0
TargetEnhancementMass2	69.0
TargetEnhancementMass3	1.75
Survey Use Tune Page CV	NO
Cone (V)	35.0
[PRODUCT IONS]	
Use High CE Product Ions Mass List File	NO
High CE Product Ions Mass List Filename	
Product Ions Match Logic	NO
Product Ions Switch Threshold (Intensity/s)	10.0
Product Ions Switch Detection Window +/- (mDa)	100.0
Product Ions Retention Time Window +/- (sec)	10.0
[NEUTRAL LOSS]	
Use Neutral Loss Mass List File	NO
Neutral Loss Mass List Filename	
Neutral Loss Match Logic	OR
Neutral Loss Switch Threshold (Intensity/s)	10.0
Neutral Loss Switch Detection Window +/- (mDa)	100.0
[MS/MS]	
MSMS Start Mass	50.0
MSMS End Mass	2000.0
Number of components	0
Use MSMS to MS Switch After Time	NO
MSMS Switch After Time (sec)	10.0
Absence of Neutral Loss	NO
Absence of Product Ion	NO
MSMS Scan Time (sec)	1.0
MSMS Interscan Time (sec)	0.0
MSMS Data Format	Continuum
Use Tune Page Cone Voltage	YES
Use MS/MS ipr File	NO
Instrument Parameter Filename [PEAK DETECTION]	

Peak Detection Window	1.0
Use Intensity based Peak Detection	YES
Charge State Tolerance Window	0.2
Charge State Extraction Window	4.0
Deisotope Tolerance Window	0.2
Deisotope Extraction Window	4.0
Discard survey data [COLLISION ENERGY]	NO
Using Auto Trap MS Collision Energy (eV)	4.000000
Using Auto Transfer MS Collision Energy (eV) [INCLUDE]	2.000000
Precursor Selection [EXCLUDE]	Everything
Use Exclude Masses List	NO
Exclude Mass Range	
Use Exclude File Masses	NO
Exclude Mass Filename	
Exclude Window +/- (mDa)	100.0
Exclude Retention Time Window [MOBILITY]	10.0
Use Precursor Drift Times	NO
Maintain Mobility Separation	YES
Override IMS Wave Velocity	YES
Override Transfer Wave Velocity	YES
IMS Wave Velocity	1000.0
Transfer Wave Velocity	175.0
Reference Centroid Average	
Reference Frequency	0.0
Reference Cone Voltage	0.0
Calibration	Dynamic 2

Function Parameters - Function 3 - TOF PARENT FUNCTION [ACQUISITION]

Survey Start Time	5.0
Survey End Time	125.0
Survey Ion Mode	ES Mode
Survey Polarity	Positive
[PARENT MS SURVEY]	
Survey Start Mass	50.0
Survey End Mass	2000.0
Parent Survey High CE (V)	30.0
TIC Threshold	5.0
Survey Scan Time	0.8
Survey Interscan Time	0.1

Survey Data Format	Continuum
Analyser	Resolution Mode
ADC Sample Frequency (GHz)	3.0
TargetEnhancementMass2	69.0
TargetEnhancementMass3	1.75
Survey Use Tune Page CV [PRODUCT IONS]	YES
Use High CE Product Ions Mass List File	NO
High CE Product Ions Mass List Filename	
Product Ions Match Logic	NO
Product Ions Switch Threshold (Intensity/s)	10.0
Product Ions Switch Detection Window +/- (mDa)	100.0
Product Ions Retention Time Window +/- (sec) [NEUTRAL LOSS]	10.0
Use Neutral Loss Mass List File	NO
Neutral Loss Mass List Filename	
Neutral Loss Match Logic	OR
Neutral Loss Switch Threshold (Intensity/s)	10.0
Neutral Loss Switch Detection Window +/- (mDa) [MS/MS]	100.0
MSMS Start Mass	100.0
MSMS End Mass	1500.0
Number of components	1
Use MSMS to MS Switch After Time	NO
MSMS Switch After Time (sec)	10.0
Absence of Neutral Loss	NO
Absence of Product Ion	NO
MSMS Scan Time (sec)	1.0
MSMS Interscan Time (sec)	0.1
MSMS Data Format	Continuum
Use Tune Page Cone Voltage	YES
Use MS/MS ipr File	NO
Instrument Parameter Filename [PEAK DETECTION]	
Peak Detection Window	1.0
Use Intensity based Peak Detection	YES
Charge State Tolerance Window	0.2
Charge State Extraction Window	4.0
Deisotope Tolerance Window	0.2
Deisotope Extraction Window	4.0
Discard survey data [COLLISION ENERGY]	NO
Using Auto Trap MS Collision Energy (eV)	4.000000

Using Auto Transfer MS Collision Energy (eV)	2.000000
[INCLUDE]	
Precursor Selection [EXCLUDE]	Everything
Use Exclude Masses List	NO
Exclude Mass Range	
Use Exclude File Masses	NO
Exclude Mass Filename	
Exclude Window +/- (mDa)	100.0
Exclude Retention Time Window [MOBILITY]	10.0
Use Precursor Drift Times	NO
Maintain Mobility Separation	YES
Override IMS Wave Velocity	YES
Override Transfer Wave Velocity	YES
IMS Wave Velocity	1000.0
Transfer Wave Velocity	175.0
Reference Centroid Average	
Reference Frequency	0.0
Reference Cone Voltage	0.0
Calibration	Dynamic 2

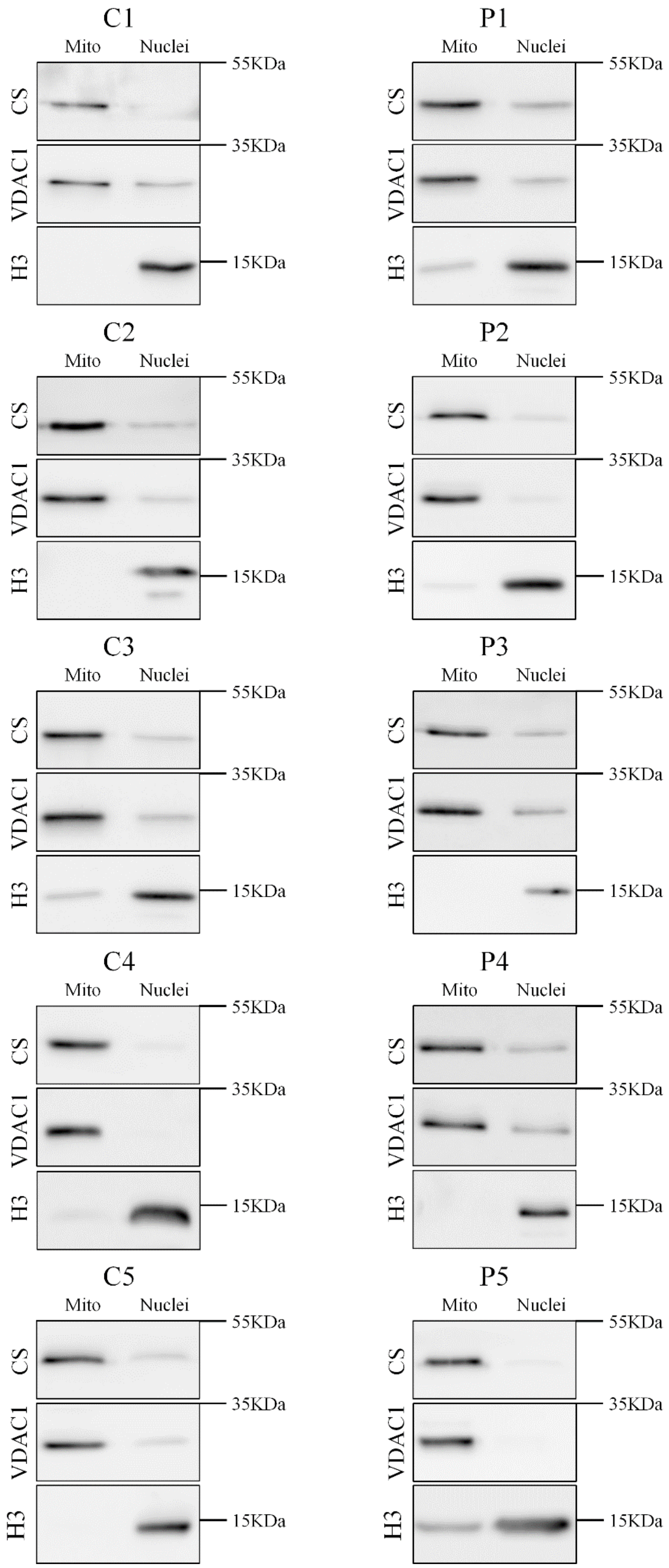

Supplementary Figure 1. Mitochondrial enrichment. Representative Western blot of VDAC1, CS and H3 proteins. *PARK2*-mutated patients (P1, P2, P3, P4 and P5); control subjects (C1, C2, C3, C4 and C5). Mitochondrial markers (VDAC1 and CS) are enriched in the mitochondrial fractions, while the nuclear marker (histone H3) is detected only in the nuclear fractions.

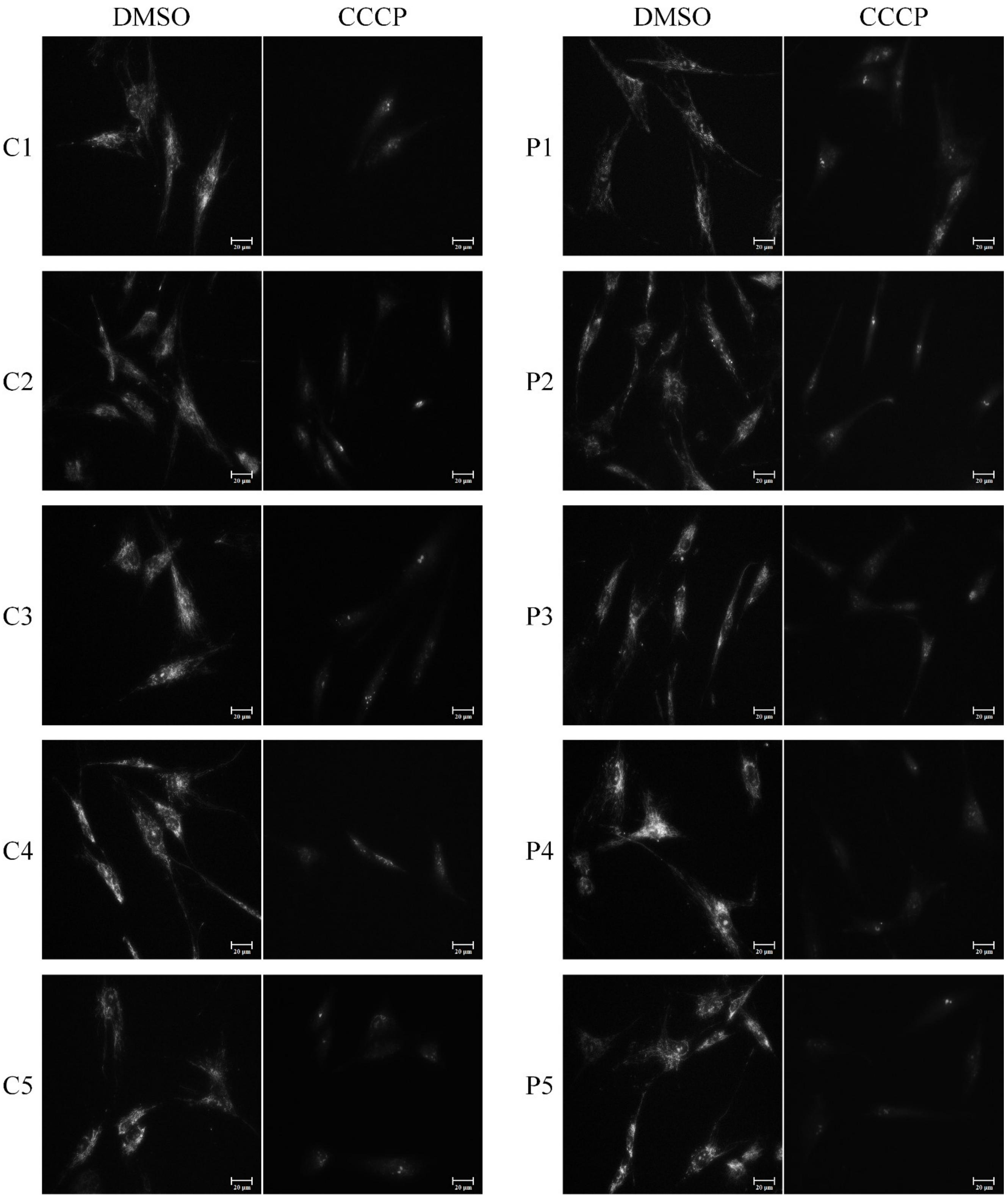

Supplementary Figure 2. Mitochondrial membrane potential. Representative images of fibroblast cells from five *PARK2*-mutated patients (P1, P2, P3, P4 and P5) and five control subjects (C1, C2, C3, C4 and C5) stained with Mitotracker Red CMXRos after DMSO or CCCP treatment.

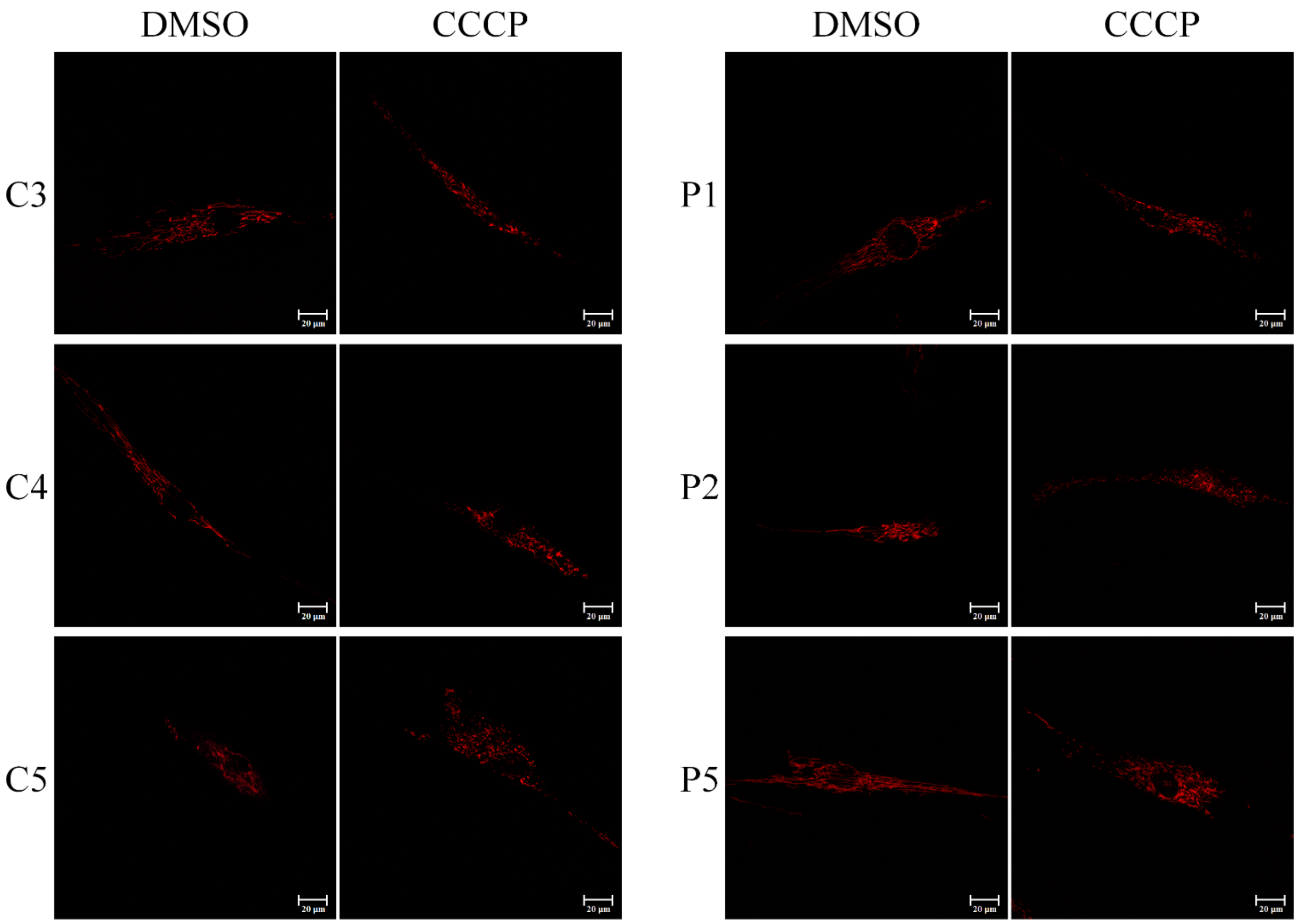

Supplementary Figure 3. Mitochondrial network. Representative immunofluorescence images of primary skin fibroblasts from three *PARK2*-mutated patients (P1, P2 and P5) and three control subjects (C3, C4 and C5) labeled with ATP synthase β antibody after DMSO or CCCP treatment.

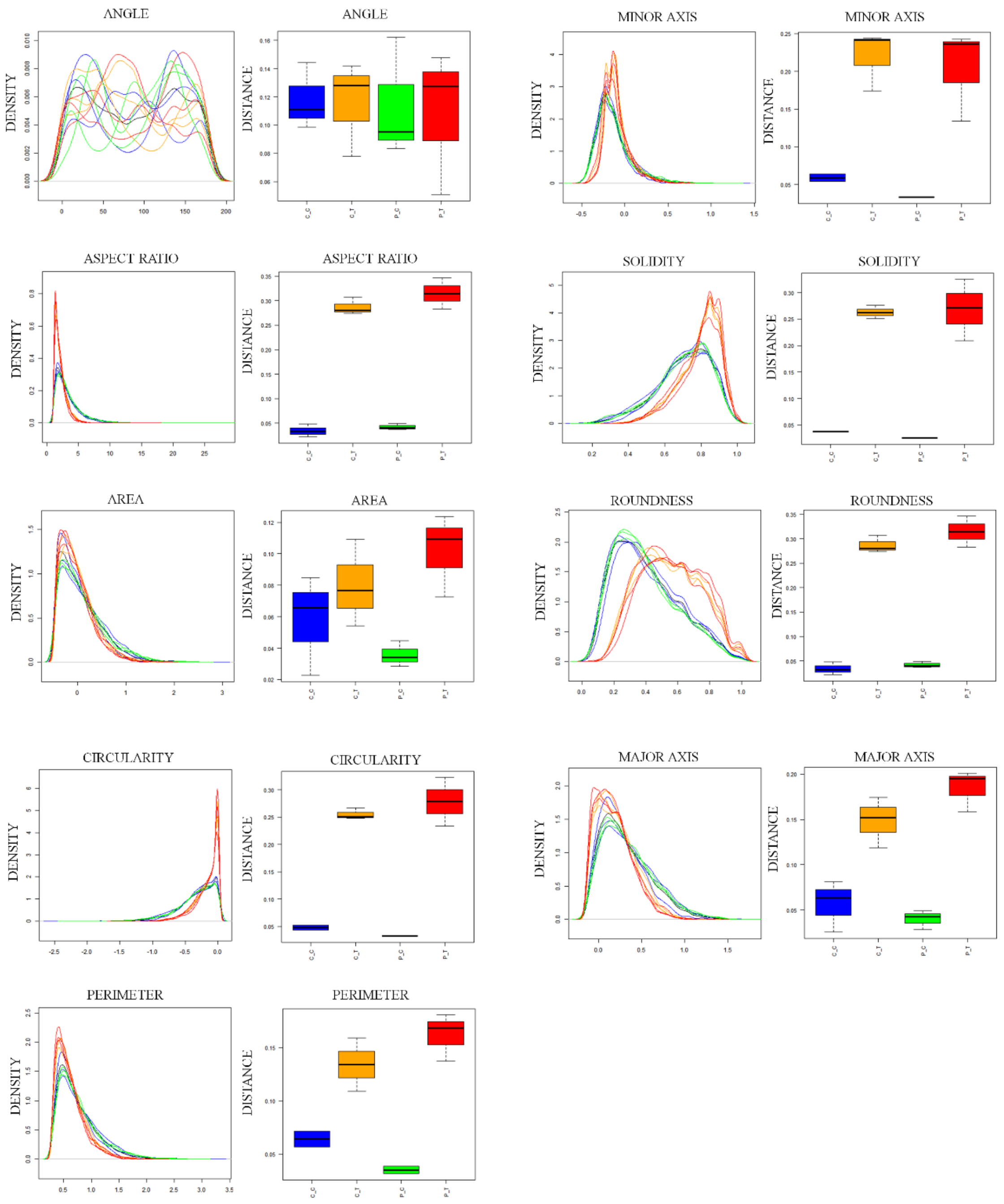

Supplementary Figure 4. Morphological network parameters. Distribution density for all particles analyzed using the "Analyze Particles" tool in five field of view from each subject and Kolmogorov-Smirnov distances of all parameters (blue: CTRL subjects/DMSO; orange: CTRL/subjects CCCP; green *PARK2*-mutated patients/DMSO; red: *PARK2*-mutated patients/CCCP).

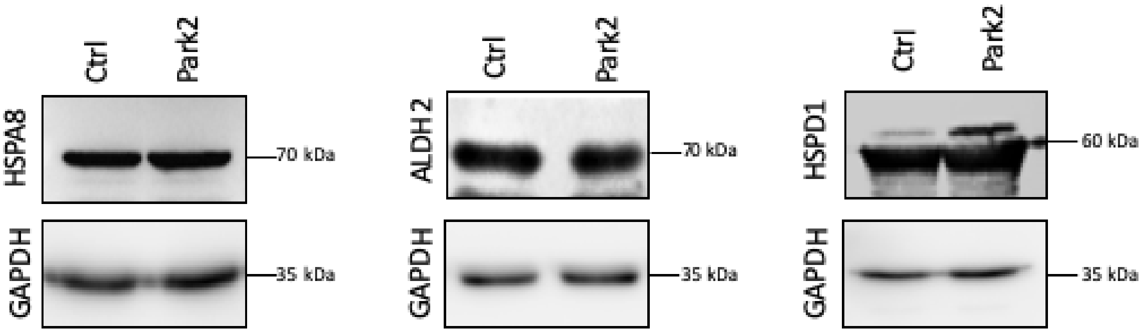

Supplementary Figure 5. Inputs of the co-IP experiment. Western blotting of the three prey proteins HSPA8, HSPD1, and ALDH2 in the mitochondrial pellets obtained from fibroblasts of C4 control subject and P3 *PARK2*-mutated subject , before performing the co-IP experiment (inputs).

Supplementary Table 1. List of 649 proteins identified in the mitochondrial fractions by shotgun proteomics showing a finite ratio.

Supplementary Table 2. List of 1457 proteins identified in the total fractions by shotgun proteomics showing a finite ratio.

Supplementary Table 3. Summary of 227 significantly up- and down- regulated and unique proteins in the mitochondrial fractions used for the enrichment analysis.

Supplementary Table 4. Summary of 168 significantly up- and down- regulated and unique proteins in the total fractions used for the enrichment analysis.

Supplementary Table 5. Summary of protein pairs that have been shown to interact in literature in at least two papers.

Supplementary Table 6. Summary of all identified enriched pathways in the mitochondrial fractions using the GSEA approach.

Supplementary Table 7. Summary of all identified enriched pathways in the total fractions using the GSEA approach.
